# Peli1 Contributions in Microglial Activation, Neuroinflammatory Responses and Neurological Deficits Following Experimental Subarachnoid Hemorrhage

**DOI:** 10.3389/fnmol.2017.00398

**Published:** 2017-11-30

**Authors:** Xue-Ping Huang, Jian-Hua Peng, Jin-Wei Pang, Xiao-Cui Tian, Xin-Shen Li, Yue Wu, Yong Li, Yong Jiang, Xiao-Chuan Sun

**Affiliations:** ^1^Department of Neurosurgery, The First Affiliated Hospital of Chongqing Medical University, Chongqing, China; ^2^Department of Neurosurgery, The Affiliated Hospital of Southwest Medical University, Luzhou, China; ^3^Department of Chongqing Key Laboratory of Biochemistry and Molecular Pharmacology, College of Pharmacy, Chongqing Medical University, Chongqing, China

**Keywords:** Peli1, subarachnoid hemorrhage, early brain injury, microglia, neuroinflammation

## Abstract

Early brain injury (EBI) following subarachnoid hemorrhage (SAH) is closely associated with neuroinflammation. Microglial activation is an early event that leads to neuroinflammation after SAH. Peli1 is an E3 ubiquitin ligase that mediates the induction of pro-inflammatory cytokines in microglia. Here we report Peli1 contributions in SAH mediated brain pathology. An SAH model was induced by endovascular perforation in adult male C57BL/6J mice. Peli1 was markedly induced in mice brains in a time-dependent manner and was predominantly expressed in CD16/32-positive microglia after SAH. Using genetic approaches, we demonstrated that decreased Peli1 significantly improved neurological deficits, attenuated brain edema, reduced over-expression of pro-inflammatory cytokine IL-6 and modified apoptotic/antiapoptotic biomarkers. In addition, Peli1 downregulation suppressed ERK and JNK phosphorylation levels via the downregulation of cIAP1/2 expression, subsequently reducing inducible nitric oxide synthase (iNOS) expression after SAH. Therefore, these findings demonstrate that Peli1 contributes to microglia-mediated neuroinflammation in EBI by mediating cIAP1/2 activation, thus promoting the activation of MyD88-dependent MAPK pathway after experimental SAH. Our findings also showed that Peli1 could promote the expression of M1 microglia polarization biomarker CD16/32 and iNOS after SAH. Targeting Peli1 exerts neuroprotective effects during EBI after SAH, thus could provide potential option for prevention-therapy in high-risk individuals.

## Introduction

Subarachnoid hemorrhage (SAH) is a devastating cerebrovascular disease that accounts for 5% of all strokes (Chen et al., 2014). Due to its high rate of mortality and morbidity, the therapeutic interventions of SAH remain a major clinical challenge faced by physicians. In past decades, cerebral vasospasm occurring between day 4 and 10 after SAH was considered as the most crucial cause of poor outcome (Pluta et al., 2009). However, the reversal of vasospasm failed to improve the outcome of SAH in multicenter clinical trials. Early brain injury (EBI), which starts in the first 72 h following aneurysmal subarachnoid hemorrhage (aSAH), has recently been considered a primary cause of mortality and disability (Helbok et al., 2015). It has been demonstrated that a complex series of pathophysiological events are involved in the development of EBI, including brain edema, neuronal cell death and blood-brain barrier (BBB) disruption (Cahill et al., 2006). In addition, accumulated evidence has highlighted a strong contribution of neuroinflammation to EBI after SAH (Sabri et al., 2013). Microglia are the resident immune cells in the central nervous system (CNS), and the effects of microglial activation related to neuroinflammation in CNS disorders are under intense scrutiny (Zhang et al., 2015). Therefore, targeting microglial activation and neuroinflammation is a promising therapeutic strategy to improve neurological deficits.

Peli (also called Pellino) was originally identified in *Drosophila* as an adaptor protein interacting with Pelle, an ortholog of the *Drosophila* homolog of mammalian interleukin-1 receptor-associated kinase (IRAK; Grosshans et al., 1999). There are three mammalian Peli members, Peli1, Peli2 and Peli3, which share strong sequence homology, expressing C-terminal RING-like domains responsible for E3 polyubiquitin ligase activity (Schauvliege et al., 2007). Peli proteins function as E3 ubiquitin ligases that interact with multiple intermediates, including IRAK1, IRAK4, TAK1 and TRAF6 (Medvedev et al., 2015). However, specificity in interactions have been found in different members of the Peli family: e.g., Peli1 and Peli2 bind IRAK1, but not Peli3 (Schauvliege et al., 2006). *In vitro* studies suggest that Peli proteins can regulate Toll-like receptor (TLR), IL-1 receptor, T-cell receptor (TCR) and TNFR signaling (Schauvliege et al., 2007; Chang et al., 2011; Yang et al., 2013; Murphy et al., 2015). Briefly, Peli1 is abundantly expressed in microglia relative to other family members. Peli1 has been suggested to have a microglial cell-specific role in mediating TLR-induced pro-inflammatory cytokine and chemokine expression (Xiao et al., 2013). Peli1 seems to positively regulate TLR2 and TLR4 signaling with both MyD88- and TRIF-dependent signaling pathways (Murphy et al., 2015). Peli1 also functions as a critical mediator for mitogen-activated protein kinase (MAPK) activation in the MyD88-dependent pathway through K63 ubiquitination of cellular inhibitor of apoptosis proteins (cIAP; Tseng et al., 2010; Hu and Sun, 2016). These findings have indicated a potential role of Peli1 in neuroinflammation. However, whether Peli1 regulates the MyD88-dependent MAPK pathway in pathological processes of SAH remains elusive.

In the present study, we show the pivotal role of Peli1 in regulating microglial activation and facilitating neuroinflammation after experimental SAH. We observed a robust induction of Peli1 expression in microglia of mouse brain after SAH. We provide molecular evidence that Peli1 regulates c-IAP1/2 signaling pathways, thereby controlling MyD88-mediated MAPK activation and induction of pro-inflammatory cytokines. Thus, targeting Peli1 may alleviate EBI, providing a novel strategy for SAH treatment.

## Materials and Methods

### Experimental Animals

All animal studies were approved by the Chongqing Medical University Biomedical Ethics Committee in Chongqing, China. All experimental procedures were performed in accordance with the National Institutes of Health guidelines for the Care and Use of Laboratory Animals. The 10–12-week-old male C57BL/6J mice were purchased from the Laboratory Animal Center of Chongqing Medical University. All mice were maintained with controlled light (12 h light/dark) under the same temperature and humidity, with food and water available *ad libitum*. A total of 118 mice were used in the current study.

### Experimental SAH Model

The SAH model was induced by endovascular perforation as previously described (Tang et al., 2015). The animals were anesthetized with an intraperitoneal injection of sodium pentobarbital (50 mg/kg). After exposure of the right common carotid artery (CCA), external carotid artery (ECA) and internal carotid artery (ICA), the ECA was ligated and transected with a 3-mm stump. A 5–0 Prolene filament (Ethicon, Somerville, NJ, USA) was advanced into the ICA through the ECA until subtle resistance and then was pushed 2 mm further to perforate bifurcation of the anterior and the middle cerebral artery. Subsequently, the filament was withdrawn. In the sham-operation group, filaments were advanced without arterial perforation.

### Experimental Design

The *in vivo* experiments were designed as follows:

#### Experiment 1

Male mice were divided into five groups at random: Sham (*n* = 3), SAH 6 h (*n* = 3), SAH 24 h (*n* = 3), SAH 48 h (*n* = 3) and SAH 72 h (*n* = 3). Animals with a SAH Grade score of less than eight were excluded from the study. The expression and time course of Peli1, cIAP1/2 and TLR4 were measured by western blot.

#### Experiment 2

Male mice were divided into two groups at random: Sham (*n* = 6) and SAH 48 h (*n* = 6).

Brain samples for hematoxylin and eosin (H&E) staining and immunofluorescence were collected.

#### Experiment 3

Male mice were divided into three groups for mechanism study: group Sham received normal saline microinjection (*n* = 29), group SAH + Peli1-NC received scrambled- sequence lentivirus microinjection (*n* = 32) and group SAH + Peli1-KD received Peli1-knockdown (KD) lentivirus microinjection (*n* = 30). Neurological scores, MRI, SAH grade, enzyme-linked immunosorbent assay (ELISA) and western blot were detected at 48 h after SAH.

### Lentiviral Vector Preparation

The lentiviral vector expressing Peli1-shRNA was constructed by Genechem Co., LTD. (Shanghai, China). Three shRNA sequences that target the mouse Peli1 sequence (GenBank NM_023324) were designed as follows: 5′-CGTAATGGGTTCACAGAAG-3′(KD1), 5′-CGGCAAAGATCGTGAATG T-3′ (KD2), 5′-GTACAAAGCACTATATCAA-3′ (KD3). The lentivirus with the scrambled sequence 5′-TTCTCCGAACGTGTCACGT-3′, was used as negative control (NC) throughout the study. The Peli1-shRNA lentiviral vector, together with lentiviral helper plasmid pHelper 1.0 and pHelper 2.0, were co-transfected into 293T-cells using Lipofectamine 2000 (Invitrogen, Carlsbad, CA, USA) and prepared to a titer of 1 × 10^9^ transfection units (TU) per mL. BV2 cells, a murine microglia cell line, were transfected with recombinant Peli1-shRNA lentivirus and NC lentivirus. The Peli1 mRNA expression was detected by quantitative real-time reverse transcription-polymerase chain reaction (qRT-PCR). The most efficient recombinant Peli1-shRNA lentivirus were used in later studies.

### qRT-PCR

Total RNA was extracted from cells using Trizol (Vazyme, Nanjing, China). For quantitative detection of Peli1 mRNA, cDNA was synthesized from 2 μg of total RNA with M-MLV Reverse Transcriptase Kit (Promega, Madison, USA). Primers were synthesized by Genechem Co., LTD. (Shanghai, China). The quantitative real-time PCR was performed using SYBR Master Mixture Kit (Takara, Otsu, Shiga, Japan) on a Roche Light Cycler 480 II Real-Time PCR instrument. The following primers were used: Peli1, Forward: 5′-GAACGTGACGGCAAAGATCG-3′, Reverse: 5′-AGGGGCTAAAGGCATGGGTG-3′; GAPDH, Forward: 5′-TGGTGAAGGTCG GTGTGAAC-3′, Reverse: 5′-GCTCCTGGAAGATGGTGATGG-3′. The mRNA levels of the Pei1 expression were normalized to GAPDH.

### Administration of Lentiviral Vector

The lateral ventricle injection of lentiviral vector was performed as previously described (Xing et al., 2012; Hironaka et al., 2015). Mice were anesthetized by intraperitoneal injection of sodium pentobarbital (50 mg/kg) and then mounted on a stereotactic frame. The skin above the skull was incised and a small hole in the skull was opened over the injection site using a microdrill. Stereotaxic coordinates were 0.4 mm posterior and 1.0 mm lateral to bregma and 3.0 mm below the dura. The 20 μL (10 μL per side) of lentivirus solution were manually injected into the lateral ventricles at a flow rate of 1 μL per minute over a period of 10 min with a microsyringe. The needle was left in place for 10 min prior to withdrawal, allowing the lentivirus solution to diffuse into the CSF. Five days after lentivirus injection, the SAH model was induced.

### Neurological Scores

Neurological deficits were evaluated 48 h after SAH using a modified Garcia’s method (Garcia et al., 1995). The assessment including spontaneous activity, symmetry in the movement of four limbs, forepaw outstretching, climbing, body proprioception and response to whisker stimulation (3–18 points) was performed. The mean neurologic scores were evaluated by two investigators who were blinded to the experimental treatment group of the animals.

### Assessment of SAH Grade

The severity of SAH was blindly assessed on the basis of an 18-point grading system as previously described (Sugawara et al., 2008). The basal brain was divided into six segments, with each segment scored from 0 to 3 according to the amount of blood clot. A total score ranging from 0 to 18 was calculated after summing the scores of each segment. Animals with a score of less than 8 were excluded from the study.

### MRI Measurement of Lesion Volume

MRI was performed 48 h after SAH using a 7.0-T MR scanner (Bruker Biospec, Ettlingen, Germany) with acquisition of T2 imaging. MRI settings were as follows: Turbo-RARE sequence, repeating time/echo time (TR/TE) = 4000/45 ms, field of view (FOV) = 2.5 cm and 20 coronal slices (0.5 mm thick). The volume of T2 hyperintensity in the white matter and ventricular volume were calculated as previously described (Okubo et al., 2013; Egashira et al., 2014).

### Hematoxylin and Eosin (H&E) Staining

The isolated mouse brain samples were fixed with 4% paraformaldehyde for 24 h at room temperature, and dehydrated through a graded alcohol series of 60, 70, 80, 90, 95 and 100% ethanol and finally embedded in paraffin. The prepared 5-μm-thick paraffin sections were deparaffinized with xylene and rehydrated. The paraffin sections were then stained using filtered Mayer’s hematoxylin (Sigma, St. Louis, MO, USA) and counterstained with eosin. The sections were dehydrated through a graded ethanol series, cleared in xylene and then mounted with mounting medium. The stained sections were examined under a light microscopy (Eclipse 55i, Nikon, Tokyo, Japan) at 400× magnification.

### Western Blot

The frozen right hemispheres of mice (perforation side) were mechanically homogenized in ice-cold radioimmunoprecipitation assay (RIPA) buffer (Boster, Wuhan, Hubei, China). The tissue lysates were centrifuged at 12,000 rpm for 15 min at 4°C and the supernatants were stored at −80°C. The protein extracts were fractionated by electrophoresis on sodium dodecyl sulfate-polyacrylamide gel and transferred to polyvinylidene difluoride membranes (Millipore, Bedford, MA, USA). The membranes were blocked in Tris-buffered saline Tween-20 (TBST) containing 5% bovine serum albumin for 2 h at room temperature and incubated overnight at 4°C with primary antibodies: anti-Peli1 (1:500, Abcam, Cambridge, UK), anti-TLR4 (1:1000, Abcam, Cambridge, UK), anti-cIAP1/2 (1:500, Abcam, Cambridge, UK), anti-Bax (1:500, CST, Danvers, MA, USA), anti-Bcl-2 (1:500, CST, Danvers, MA, USA), anti-iNOS (1:500, Proteintech, Wuhan, Hubei, China), anti-p-JNK (CST, Danvers, MA, USA), anti-p-ERK (1:1000, Santa Cruz, Dallas, TX, USA), anti-MyD88 (1:500, Proteintech, Wuhan, Hubei, China), anti-GAPDH (1:1000, Santa Cruz, Dallas, TX, USA). GAPDH was used as an internal loading control. Appropriate secondary antibodies conjugated with horseradish peroxidase were incubated with the membrane for 1 h at room temperature. The immune bands were detected with an ECL kit (Advansta, Menlo Park, CA, USA). The density of each band was quantified using Image Lab software (Bio-Rad, Hercules, CA, USA).

### Immunofluorescence

Forty-eight hours after SAH, mice were anesthetized and perfused transcardially with phosphate-buffered solution (PBS) and with 4% paraformaldehyde. The brains were rapidly isolated and post-fixed in 4% paraformaldehyde for 4 h, followed by overnight immersion in phosphate buffer containing 30% sucrose. The brains were then embedded in OCT solution, and 8-μm coronal brain cryosections were prepared. Sections were fixed in ice-cold acetone and blocked with 10% goat serum for 2 h. Next, the sections were incubated at 4°C overnight with the following primary antibodies: rat anti-CD16/32 (1:150, BD Pharmingen, San Diego, CA, USA), rabbit anti-Peli1 (1:250, Abcam, Cambridge, UK). After being washed in PBS, the sections were incubated with appropriate secondary antibody for 30 min at 37°C: TRITC-conjugated goat anti-rat IgG (1:200, Proteintech, Wuhan, Hubei, China) and DyLight 488-conjugated goat anti-rabbit IgG (1:200, Abbkine, California, USA). The nuclei were stained with DAPI. The stained sections were visualized with a fluorescence microscope (Nikon, Tokyo, Japan).

### ELISA

IL-6 levels of animals were measured by ELISA. The frozen brain samples were mechanically homogenized and centrifuged at 12,000 rpm for 15 min at 4°C. The protein content was detected using a bicinchoninic acid assay kit (Bio-Rad Laboratories, Hercules, CA, USA). The levels of IL-6 were quantified using ELISA kits for mouse according to the manufacturer’s instructions (R&D, Minneapolis, MN, USA).

### Statistical Analysis

The SPSS software 21.0 (SPSS Inc., Chicago, IL, USA) was used for statistical analyses. All data were presented as mean ± SD and subjected to one-way analysis of variance (ANOVA) followed by the LSD or Dunnett’s T3 *post hoc* test. *P* < 0.05 was accepted as statistically significant.

## Results

### Peli1 Is Upregulated and Involved in TLR4-Dependent Microglial Activation after SAH

**Figure 1:**
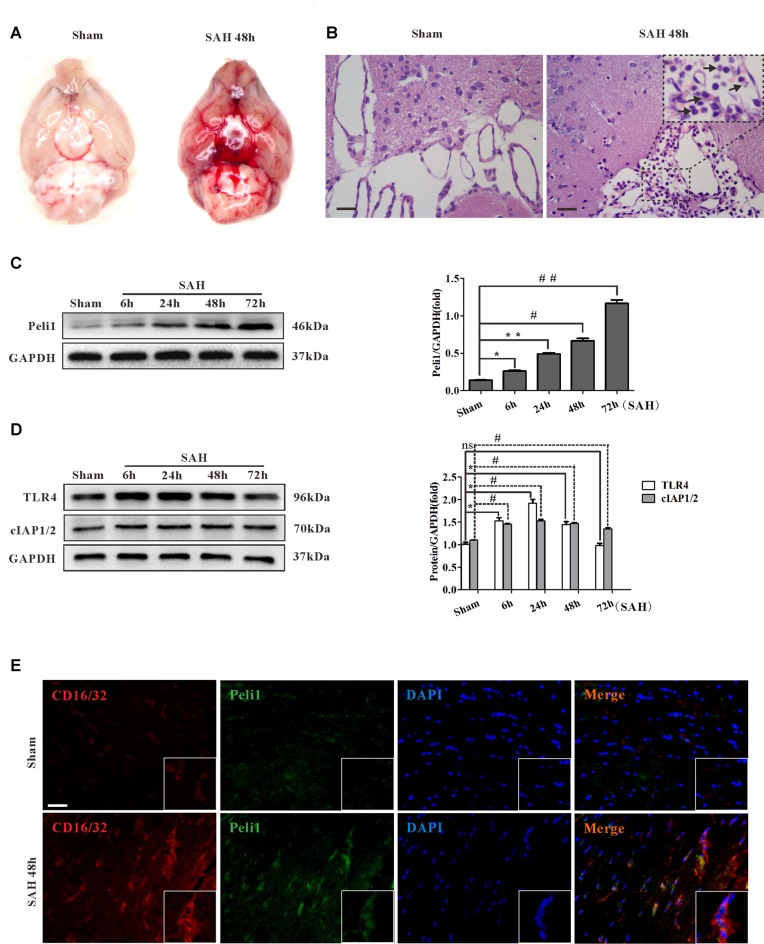
Peli1 is upregulated and mediates microglial activation in subarachnoid hemorrhage (SAH) model. **(A)** A mass of blood clots were particularly pronounced around arterial circle of Willis after SAH. **(B)** Hematoxylin & eosin (H&E) staining of coronal section from mice subjected to sham and SAH on 48 h for visualizing myeloid cells infiltration in subarachnoid layers (arrows). Original magnification is ×400. Scale bars are 50μm. **(C)** Western blot showing higher levels of Peli1 protein expression at 6, 24, 48 and 72 h after SAH (left). Quantification (right) of normalized Peli1 levels were shown (*n* = 3 per time point, *P = 0.009, **P < 0.001, ^#^*P* < 0.001, ^##^*P* < 0.001 vs. Sham). **(D)** Left: Western blot showing TLR4 and cIAP1/2 expression at the indicated time points after SAH. Right: quantification of normalized TLR4 and cIAP1/2 levels (*n* = 3 per time point, *P < 0.01, ^#^*P* < 0.001, ns indicating non-significant, vs. Sham group). **(E)** Immunofluorescent staining for CD16/32 (red) reflecting co-localization of Peli1 (green) by activated microglia in mouse brains 48 h after SAH and sham-operated group. Nuclei were stained blue by DAPI. Original magnification is ×400. Scale bars are 50 μm. The experiments were repeated three times.

Experimental models of SAH in mice were induced by endovascular perforation of the bifurcation of the right middle cerebral artery and anterior artery. There was no blood clot in sham-operated group. Vessel perforation resulted in a significant extravasation of blood into the subarachnoid space (Figure 1A). In the H&E staining, blood was present in the subarachnoid layer of animals subjected to SAH but not in sham animals. Myeloid cells were found to be recruited into the subarachnoid layers of mice subjected to SAH after 48 h but not in sham animals (Figure 1B).

To investigate the role of Peli1 in SAH, we analyzed Peli1 expression in mouse cerebral tissues by western blot at 6, 24, 48 and 72 h after SAH. Interestingly, we observed a time-dependent increase in Peli1 protein expression after experimental SAH (Figure 1C). Furthermore, western blot analysis for Peli1 protein expression in different types of CNS cell lines was used to find the cell sources of Peli1. In line with previous findings (Xiao et al., 2013), Peli1 was predominantly expressed in microglia, whereas it was lowly expressed in neurons, astrocytes and oligodendrocyte precursor cells (OPCs; Supplementary Figures S1A,B). We next studied expression and localization of Peli1 in microglia by double-immunofluorescence staining of Peli1 and an activated microglia-specific marker, CD16/32. The induction of SAH resulted in a robust increase in cytoplasmic Peli1 expression and cytomembranous CD16/32 expression in activated microglia (Figure 1E). These results indicate that increased Peli1 primarily exists in microglia and is associated with microglial activation after SAH.

Recent studies suggest that the activation of TLR4 signaling pathway is necessary for microglial activation (Bell et al., 2013; Hanafy, 2013; Wu et al., 2017). To further elucidate relationship between Peli1 and TLR4 signaling, we analyzed the expression of TLR4 and Peli1-associated regulator at different time points after SAH. Slightly different from other observations (Ma et al., 2009), western blot analysis revealed sharply increased TLR4 protein expression at the earliest 6 h after SAH, further reaching a peak at 24 h after SAH (Figure 1D). The TLR4 expression recovered partially at 48 h after SAH, and was equivalent to the sham-operated group at 72 h after SAH. It was proposed that cIAP1/2 may be recruited to MyD88 dependent TLR4, thus promoting MAPK activation and induction of inflammatory cytokines (Tseng et al., 2010). We examined cIAP1/2 protein expression in SAH models. The result of western blot analysis showed that cIAP1/2 protein levels were increased from 6 h to 24 h after SAH, recovered at 48 h, and remained at a high level until 72 h after SAH (Figure 1D).

### Knockdown of Peli1 Improves Neurological Outcomes and Reduces Cerebral Edema after SAH

On the basis of observation that Peli1 was upregulated following SAH stimuli, we hypothesized that Peli1 may play a role in cerebral damage during SAH. To elucidate the direct effects of Peli1 expression on SAH outcome, we suppressed expression of Peli1, using a lentiviral vector constructs expressing shRNA targeting Peli1. In order to establish the efficiency of Peli1-shRNA lentiviral vector, we first examined the expression of Peli1 mRNA in the microglia cell line, BV2 cells. The mRNA levels of Peli1 were downregulated by 47.5% (KD1), 79.4% (KD2) and 26.3% (KD3), respectively (Supplementary Figure S2). The lentiviral vector of Peli1-KD2, showing the highest efficiency, was used for further injection into the lateral ventricles of mice.

**Figure 2:**
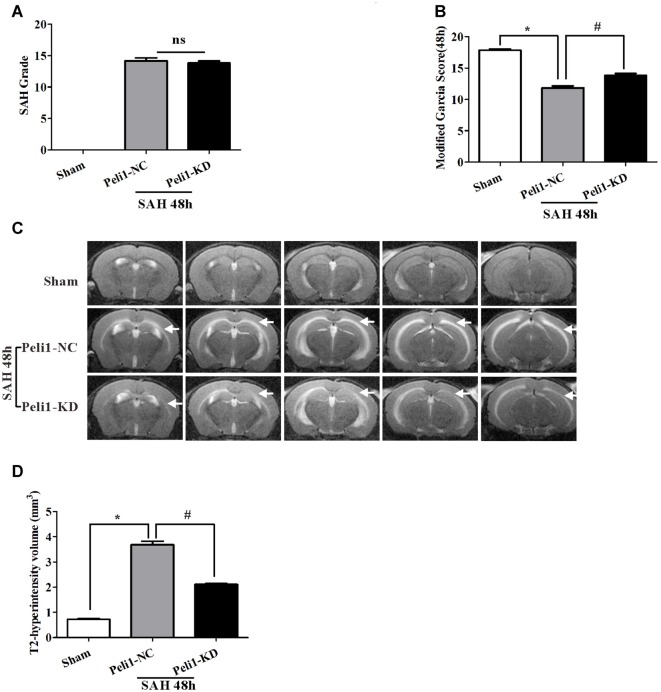
Knockdown of Peli1 alleviates neurological deficits and reduces T2 hyperintensity after SAH. **(A)** Similar SAH grades were observed in the negative control (NC) lentivirus-treated (Peli1-NC) group and Peli1-knockdown lentivirus- treated (Peli1-KD) group at 48 h after SAH (*n* = 6 per group). **(B)** The neurological scores were significantly lower in Peli1-NC group than that of sham group 48 h after SAH (*n* = 6 per group, *P < 0.001). Downregulation of Peli1 significantly improved neurologic scores compared with Peli1-NC group 48 h after SAH (*n* = 6 per group, ^#^*P* < 0.001, vs. Peli1-NC group). **(C)** Coronal T2 MRI images of sham and SAH mice brains at 48 h. T2-hyperintensity was observed with markedly increased in mice undergoing endovascular perforation compared with sham-operated group. The white arrows indicate the white matter regions. **(D)** The measured volume of T2-hyperintense region showed an increase in Peli1-NC group of animals 48 h after SAH in comparison to sham-operated animals (*n* = 3 per group, *P < 0.001). Knockdown of Peli1 significantly attenuated T2 hyperintensity compared with Peli1-NC group of animals 48 h after SAH (*n* = 3 per group, ^ #^*P* < 0.001).

The SAH grade scores were evaluated in groups of sham-operated mice, NC lentivirus-treated (Peli1-NC) mice and Peli1-knockdown lentivirus-treated (Peli1-KD) mice 48 h after SAH. The observed SAH scores were 0 in the sham group, 14.2 ± 1.2 in the SAH + Peli1-NC group, and 13.8 ± 0.8 in the SAH + Peli1-KD group (Figure 2A). No significant difference was found between the SAH groups (*P* = 0.322). The neurological scores of SAH + Peli1-NC group were significantly lower than that of the sham group, whereas knockdown of Peli1 improved neurologic scores compared with the Peli1-NC group 48 h after SAH (Figure 2B).

A markedly increased T2-hyperintensity was observed with in mice undergoing endovascular perforation (SAH) compared with the sham-operated group at 48 h (Figure 2C). The T2-hyperintense region in Peli1-NC mice reached beyond the ventricles and was prominent in white matter 48 h after SAH. However, downregulation of Peli1 significantly attenuated T2 hyperintensity, especially in white matter, in comparison to Peli1-NC mice 48 h after SAH (Figures 2C,D). These results suggest that knockdown of Peli1 could reduce cerebral edema induced by experimental SAH.

### Peli1 Ablation Attenuates Apoptosis and Production of Pro-Inflammatory Cytokines after SAH

**Figure 3:**
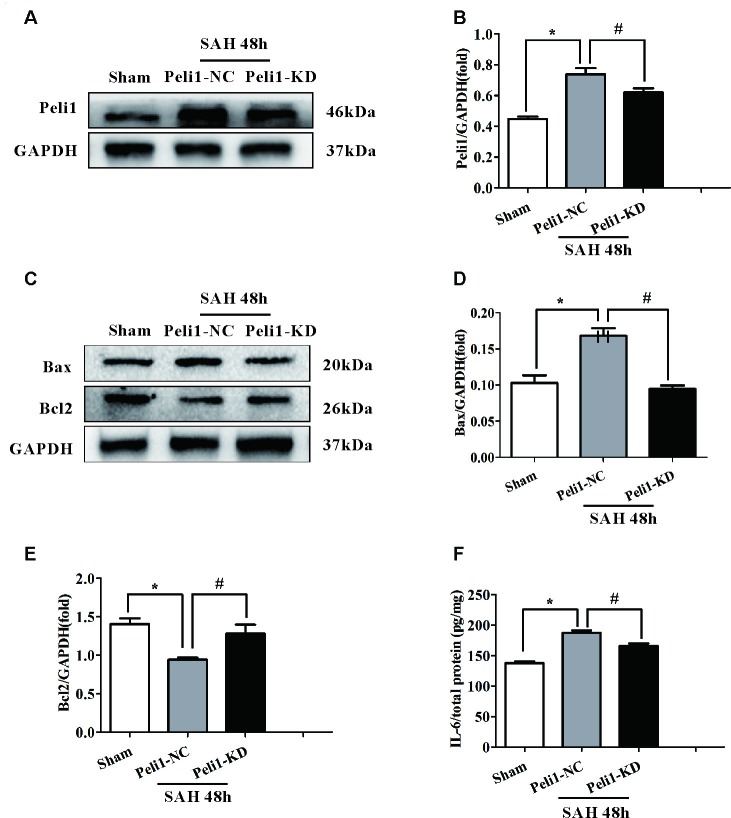
Downregulation of Peli1 attenuates apoptosis and production of pro-inflammatory cytokines after SAH. **(A)** Immunoblot analysis of Peli1 expression in sham, Peli1-NC and Peli1-KD groups of mice. **(B)** Quantification of Peli1 expression normalized to GAPDH showed that the levels of Peli1 protein were increased in Peli1-NC mice 48 h after SAH compared to sham group (*n* = 5 per group, *P < 0.001). However, administration of Peli1-KD lentiviral vectors significantly attenuated expression of Peli1 compared to Peli1-NC group 48 h after SAH (^#^*P* = 0.016). **(C–E)** Immunoblotting of apoptosis-related protein Bax and Bcl2 in the brain extracts of sham-operated mice, Peli1-NC and Peli1-KD mice 48 h after SAH. Downregulation of Peli1 significantly reduced pro-apoptotic protein Bax, and enhanced the decreased level of Bcl2. The normalized Bax expression were shown graphically in** (D)** (*n* = 5 per group, *P < 0.001 vs. sham; ^#^*P* < 0.001 vs. SAH + Peli1-NC mice). Quantifications of the normalized Bcl2 protein levels were shown in **(E)** (*n* = 5 per group, *P < 0.001 vs. sham; ^#^*P* = 0.001 vs. SAH + Peli1-NC mice). **(F)** ELISA analysis showing that downregulation of Peli1 markedly alleviated the expression of IL-6 48 h after SAH (*n* = 3, *P < 0.001 vs. sham; ^#^*P* = 0.005 vs. SAH + Peli1-NC mice).

It has previously been reported that the Peli1 is involved in regulating apoptotic pathway in microglia (Kinsella et al., 2016). However, the lack of any data explored the role of Peli1 in the apoptotic pathway after SAH. Given that apoptotic pathways are activated following the onset of SAH (Hasegawa et al., 2011), we hypothesized that Peli1 may play a role in the regulation of apoptotic pathway after SAH. First, we found that the expression of Peli1 protein was increased in Peli1-NC mice 48 h after SAH compared to sham-operated mice. However, Peli1-KD mice showed a decrease expression of Peli1 protein in comparison to Peli1-NC mice 48 h after SAH (Figures 3A,B). Compared to the sham-operated group, pro-apoptotic protein Bax was significantly increased in Peli1-NC mice 48 h after SAH, whereas it was decreased by downregulation of Peli1 48 h after SAH (Figures 3C,D). Expression of anti-apoptotic protein Bcl2 was reduced 48 h after SAH. However, knockdown of Peli1 led to partially rescue Bcl2 expression (Figures 3C,E). Taken together, these results indicate an important role of Peli1 in regulating the pro-apoptotic signaling after SAH.

Pro-inflammatory cytokines, such as IL-6 and TNF-α, have been shown to be strongly associated with EBI after SAH (Song et al., 2014). Thus, we measured IL-6 in homogenates of the ipsilateral hemisphere using ELISA. The expression of IL-6 was significantly higher in Peli1-NC mice 48 h after SAH compared with sham-operated mice (Figure 3F). Downregulation of Peli1 significantly suppressed the production of IL-6 compared with the Peli1-NC group 48 h after SAH, indicating that Peli1 acts as a positive regulator of pro-inflammatory cytokine induction.

### Decreased Peli1 Suppresses Microglial Activation by Inhibiting MAPK Signaling Pathway after SAH

**Figure 4:**
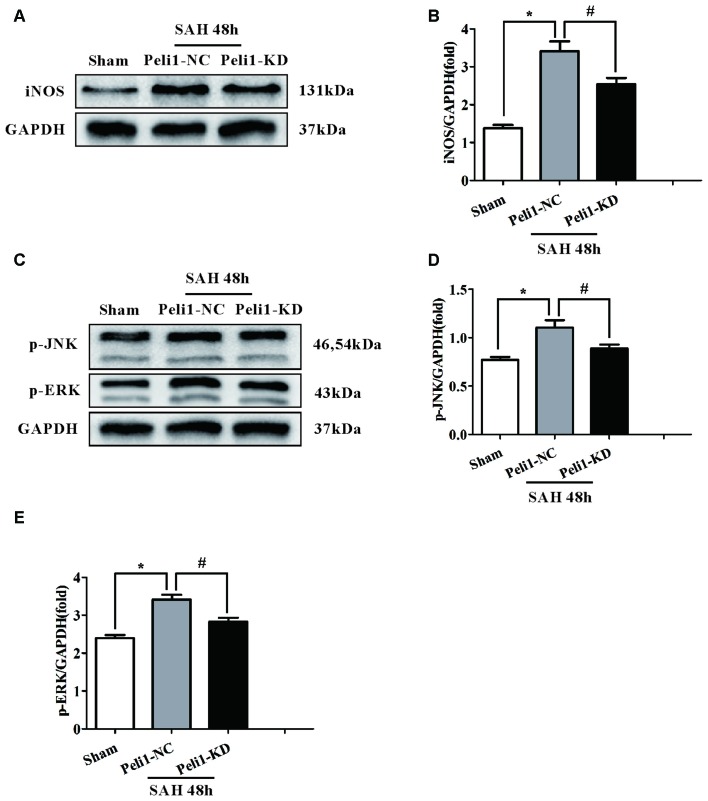
Downregulation of Peli1 inhibits inducible nitric oxide synthase (iNOS) expression and suppresses activation of MAPK signaling pathway.** (A)** Immunoblotting of iNOS was performed in brain extracts from sham- or SAH-operated Peli1-NC and Peli1-KD mice. Downregulation of Peli1 reduced the expression of iNOS 48 h after SAH. Quantification of iNOS normalized to GAPDH expression was shown in **(B)** (*n* = 5 per group, *P < 0.001 vs. sham; ^#^*P* = 0.002 vs. SAH + Peli1-NC group). **(C–E)** Peli1 activates MAPK signaling pathway. Western blot analysis of phosphorylated ERK and JNK in brain homogenates from sham- or SAH-operated Peli1-NC and Peli1-KD mice. Downregulation of Peli1 decreased phosphorylation of JNK and ERK 48 h after SAH. Quantification of normalized p-JNK was shown in **(D)** (*n* = 5 per group, *P < 0.001 vs. sham; ^#^*P* = 0.002 vs. SAH + Peli1-NC group). Quantification of normalized p-ERK was shown in **(E)** (*n* = 5 per group, *P < 0.001 vs. sham; ^#^*P* = 0.013 vs. SAH + Peli1-NC group).

It has been demonstrated that inducible nitric oxide synthase (iNOS) is a M1-type activated microglia marker (Hu et al., 2012). To elucidate the effect of Peli1 downregulation on microglial activation, we assessed the expression of iNOS in sham-operated, SAH + Peli1-NC and SAH + Peli1-KD groups by western blot. As depicted in Figures 4A,B, expression of iNOS was significantly increased in Peli1-NC mice 48 h after SAH at ~2.8-fold compared with the sham-operated group. Administration of Peli1-KD lentiviral vectors significantly reduced the expression of iNOS compared to the Peli1-NC group 48 h after SAH, suggesting that knockdown of Peli1 suppresses microglial activation.

The MAPK family members, such as ERK and JNK, are involved in controlling the production of pro-inflammatory cytokines in activated microglial cells (Santa-Cecilia et al., 2016). Therefore, we investigated whether Peli1 modulates these signaling pathways. We found that ERK and JNK phosphorylation levels were significantly increased in brain homogenates from Peli1-NC mice 48 h after SAH. This effect was dampened by knockdown of Peli1 (Figures 4C–E). Collectively, these findings demonstrate that decreased Peli-1 partially reduced microglial activation biomarker, iNOS, and differentially regulated MAPK pathway signaling molecules after SAH.

### Depletion of Peli1 Attenuates cIAP1/2 Expression after SAH

**Figure 5:**
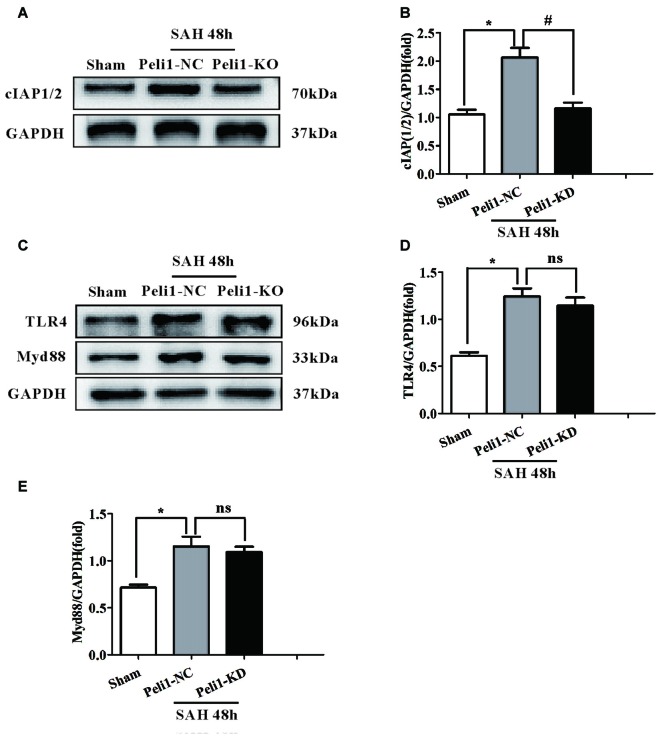
The Effects of Peli1 knockdown on cIAP1/2 expression.** (A)** Immunoblotting of cIAP1/2 expression in sham, Peli1-NC and Peli1-KD groups of mice. **(B)** Quantification of cIAP1/2 normalized to GAPDH expression showed that Peli1 enhanced cIAP1/2 expression 48 h after SAH (*n* = 5 per group, *P < 0.001 vs. sham; ^#^*P* < 0.001 vs. SAH + Peli1-NC group). **(C–E)** Downregulation of Peli1 has no effects on protein level of TLR4 and MyD88 48 h after SAH. Quantification of TLR4 normalized to GAPDH expression was shown in **(D)** (*n* = 5 per group, *P = 0.004 vs. sham; ns, *P* = 0.891, indicating non-significant, vs. SAH + Peli1-NC group). The normalized MyD88 was shown in **(E)** (*n* = 5 per group, *P = 0.006 vs. sham; ns, *P* = 0.771, indicating non-significant, vs. SAH + Peli1-NC group).

On the basis of our data and other observations that Peli1 activates the MAPK signaling pathway (Xiao et al., 2013; Li et al., 2016), we proposed that Peli1 functions as a key upstream regulator of these inflammatory pathways. We found that mice treated with SAH showed significantly increased levels of cIAP1/2 expression in comparison to sham-operated mice. However, knockdown of Peli1 reduced cIAP1/2 expression compared with Peli1-NC mice 48 h after SAH (Figures 5A,B).

As our data demonstrated that a major role of Peli1 was to activate the SAH-induced MAPK signaling pathway, which was mediated by MyD88-dependent TLR4 (Xiao et al., 2013), we asked whether Peli1 affected MyD88 and TLR4 expression. We found that the protein levels of MyD88 and TLR4 were increased 48 h after SAH, whereas there was no significant difference in MyD88 and TLR4 expression between the Peli1-NC group and Peli1-KD group (Figures 5C–E). Thus, these data indicate that Peli1 functions as a downstream target in the MyD88-dependent TLR4 pathway after SAH.

## Discussion

**Figure 6:**
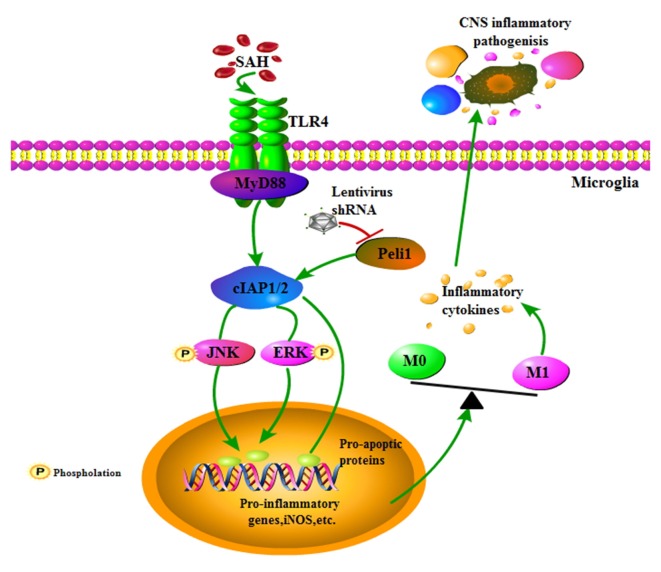
Schema depicting the Peli1-mediated pathway following SAH. TLR4 signaling is initiated by SAH stimuli. Increased Peli1 after SAH positively regulates MyD88-dependent TLR4 signaling via cIAP1/2 activation. Upregulated cIAP1/2 facilitates phosphorylation of MAPK signaling pathway and activates pro-apoptotic pathway. These combined effects promotes microglia polarization into the M1 phenotype, sequentially releasing destructive pro-inflammatory cytokines to amplify the inflammatory response in the central nervous system (CNS). Suppression of Peli1 by Peli1-shRNA lentivirus impairs the induction of pro-inflammatory cytokines.

EBI is proposed to be the primary cause of morbidity and mortality in SAH patients and is associated with neuroinflammation (Helbok et al., 2015). Microglial activation is an early event that leads to production of pro-inflammatory cytokines after SAH, and inhibition of microglial activation improves neurological outcomes in EBI (Zhang et al., 2015), emphasizing promising strategies for targeting microglial activation in the treatment of SAH. The present study revealed that the E3 ubiquitin ligase Peli1 promotes microglia-mediated neuroinflammation in EBI through activating cIAP1/2 and triggering activation of MAPK signaling pathway after SAH. Additionally, our data showed that a lentiviral vector mediated gene knockdown of Peli1 that inhibited aberrant Peli1 upregulation following SAH, provided neuroprotection against EBI (Figure 6), including attenuation of neurological deficit, brain edema, neuroinflammation and cell apoptosis. Thus, targeting Peli1 may serve as a promising alternative treatment to relieve EBI after SAH.

An increasing number of studies agree that microglia, as the resident innate immune cells in the CNS, can assume diverse phenotypes in response to different stimuli. In particular, lipopolysaccharide (LPS) and interferon-γ (IFN-γ) stimulation promote microglia polarization into the M1 phenotype. M1 microglia release destructive pro-inflammatory cytokines such as IL-6, TNF-α, iNOS, CD16 and CD32, which are involved in neuronal death and brain injury (Hu et al., 2012; Wang et al., 2016). In contrast, stimulation with IL-4 drives microglial polarization toward the M2 phenotype. M2 microglia promote arginase-1 (Arg1), and IL-10 and CD206 expression, which are related to neuroprotection (Martinez et al., 2009; Liu et al., 2013). However, the mechanisms that regulate the dual function of microglia are still missing. Therefore, an improved understanding of the microglia polarization is beneficial for advancing our knowledge of SAH mediated pathology.

Here, our results demonstrated that microglia were the major source of Peli1 expression in mice brains after SAH compared with other CNS cells such as neurons, astrocytes and oligodendrocytes. Our results are consistent with the recent report that Peli1 is predominantly expressed in microglia under the condition of inflammatory stimulation (Xiao et al., 2013). To further explore the role of Peli1 in microglia after SAH, we used double-immunofluorescence staining to show that CD16/32 expression was increased along with augmentation of Peli1 in microglia after SAH. In addition, knockdown of Peli1 markedly attenuated expression of iNOS, which is considered a pro-inflammatory protein of microglia (Wang et al., 2016). These results suggest that Peli1, which is cell type-specific, is required for microglial activation. Peli1 upregulation correlated with increased M1 microglia polarization biomarker CD16/32 after SAH.

Accumulated studies have shown that TLR4-mediated MAPK signaling pathway plays an important role in eliciting neuroinflammation after SAH (Guo et al., 2016; Han et al., 2017). It has been determined that MAPK signaling pathway was activated after SAH, leading to production of inflammatory cytokines. The activities of MyD88-dependent MAPK in microglia could be downregulated by Peli1 on experimental autoimmune encephalomyelitis (EAE; Xiao et al., 2013). In this study, we found that phosphorylated levels of ERK and JNK were increased after SAH. Downregulation of Peli1 remarkably reduced the phosphorylation of ERK and JNK, but it had no effect on MyD88 and TLR4 after SAH. The activation of MAPK signaling pathway could also aggravate EBI following SAH by inducing proapoptotic pathway (Huang et al., 2013). Our results demonstrated that Peli1 knockdown led to upregulation of Bcl2 expression and reduction of Bax expression following SAH. These results suggest that Peli1 mediates MAPK activation and acts as a proapoptotic mediator after SAH. The Bcl2 expression was partially modified when Peli1 over-expression was ameliorated following SAH, indicating that Peli1 may be involved in regulation of multiple apoptosis signaling. It has previously been reported that Peli1 is involved in apoptosis signaling via interacting with RIP1, IKK_ε_, and IRKA1 in macrophages and microglia (Chang et al., 2009; Smith et al., 2011; Medvedev et al., 2015). A recent study has shown that Peli1 facilitates K63-linked ubiquitination of the IRAK-TRAF6 complex, subsequently mediating TLR4-induced NF-κB signaling in microglia (Kinsella et al., 2016). It needs to be investigated in the future studies that whether Peli1 plays a role in TLR4-NF-κB signaling following SAH.

It is reported that Peli1 regulates the TLR pathways via ubiquitin-dependent upregulation of cIAP1/2, which is required for the activation of downstream MAPK signaling pathway in various macrophages, including microglia and Kupffer cells (Xiao et al., 2013; Li et al., 2016). Upon activation by the TLR signals, Peli1, as a E3 ligase for cIAP1/2, mediates K63-linked ubiquitination and catalytic activation of cIAP1/2 in microglia, since the Peli1 deficiency attenuated LPS-stimulated cIAP1/2 ubiquitination. Activated cIAP1/2 mediates K48 ubiquitin ligase activity and ubiquitin-dependent degradation of TRAF3, subsequently inducing activation of the MAPK signaling pathway (Xiao et al., 2013). Herein we have clearly shown a significant positive correlation between Peli1 and cIAP1/2 expression. Peli1 and cIAP1/2 expression were significantly increased following SAH. However, cIAP1/2 expression was attenuated by knockdown of Peli1 after SAH, indicating that Peli1 mediated activation of cIAP1/2. Notably, downregulation of Peli1 also led to the blockage of ERK and JNK phosphorylation. The results suggest that Peli1 mediates cIAP1/2 ubiquitination and activation, thus promoting activation of the MAPK signaling pathway after SAH.

Notably, previous studies have mainly investigated the molecular mechanism of Peli1 in chronic neuroinflammation, such as EAE (Xiao et al., 2013; Lereim et al., 2016). The current study for the first time investigated the role of Peli1 in acute neuroinflammation. However, this study has limitations in that cIAP1/2 did not directly regulate the MAPK pathway, although MAPK activation could be blocked by cIAP1/2 after SAH. Based on previous reports (Xiao et al., 2013), catalyzed cIAP1/2 conjugates K48-linked polyubiquitin chains to TRAF3, subsequently leading to TRAF3 degradation and induction of the MAPK signaling pathway in microglia after SAH. However, the mechanism by which Peli1 affects TRAF3 expression after SAH needs to be further explored. Moreover, although downregulation of Peli1 modified apoptotic/anti- apoptotic biomarkers, we must acknowledge the limitations of apoptosis analysis in methods, e.g., failing to conduct TUNEL assay. In addition, it is worth noting that polarization to the M1-type microglia is strongly correlated with white matter injury (Wang et al., 2013). In line with a recent study (Egashira et al., 2015), we observed that increased T2 hyperintensity was prominent in the white matter of cerebral regions 48 h after SAH. Intriguingly, downregulation of Peli1 significantly attenuated T2 hyperintensity, especially in the white matter following SAH, suggesting Peli1 role in regulating white matter injury after SAH. It needs to be further established whether it is a direct or an indirect effect of Peli-1 on white matter injury after SAH.

In summary, our data have demonstrated that Peli1 promotes M1 microglia polarization via a mechanism in which Peli1 functions as a downstream regulator in the MyD88-dependent TLR4 pathway to mediate cIAP1/2 activation, thus promoting activation of the MAPK pathway. Knockdown of Peli1 exerts neuroprotective effects against EBI, providing a promising therapeutic strategy for EBI after SAH.

## Author Contributions

X-PH and X-CS conceived and designed the experiments. X-PH, J-HP, J-WP and X-CT performed the experiments. X-PH and X-SL contributed to data analysis. YW and YL contributed reagents and analysis tools. X-PH and YJ drafted the manuscript. YJ and X-CS contributed to the study supervision and manuscript revision.

## Conflict of Interest Statement

The authors declare that the research was conducted in the absence of any commercial or financial relationships that could be construed as a potential conflict of interest.
