# Supplementary material for: Peli1 Contributions in Microglial Activation, Neuroinflammatory Responses and Neurological Deficits Following Experimental Subarachnoid Hemorrhage
Source: Front Mol Neurosci. 2017 Nov 30;10:398. doi: 10.3389/fnmol.2017.00398 (PMC5714869; doi:10.3389/fnmol.2017.00398)
Supplement: Supplementary file 1 [file Data_Sheet_1.docx]

**Supplemental Methods**

**Cell Lines**

The HT22 hippocampal neuronal cell line, mouse astrocytes (MA), mouse oligodendrocyte precursor cells (MOPC) and BV-2 microgia cell line were used.

**Western Blot**

The collected cells were washed gently with PBS and were mechanically homogenized in ice-cold radioimmunoprecipitation assay (RIPA) buffer (Boster, Wuhan, China). The protein concentration of supernatants were determined using a bicinchoninic acid assay kit (Bio-Rad Laboratories, Hercules, USA). The protein extracts were fractionated by electrophoresis on sodium dodecyl sulfate-polyacrylamide gel and transferred to polyvinylidene difluoride membranes (Millipore, MA, USA). The membranes were blocked in Tris-buffered saline Tween-20 (TBST) containing 5% bovine serum albumin for 2 hours at room temperature and incubated overnight at 4 °C with primary antibodies: anti-Peli1 (1:500, Abcam, Cambridge, UK) and anti-GAPDH (1:1000, Santa Cruz, Dallas, USA). GAPDH was used as an internal loading control. Appropriate secondary antibodies conjugated with horseradish peroxidase were incubated with the membrane for 1 hour at room temperature. The immune bands were detected with ECL kit (Advansta, CA, USA). The density of each band was quantified using Image Lab software (Bio-Rad, Hercules, USA).

**Supplementary Figure Legends**

**SUPPLEMENTARY FIGURE S1** Peli1 is highly expressed in microglia. **(A)** Western blot analysis of Peli1 expression in different cell lines. **(B)** Quantification of normalized Peli1 levels were shown (n=3 replicates, * *P*<0.05 vs. HT22, MA and MOPC cells).

**SUPPLEMENTARY FIGURE S2** Knockdown of Peli1 mRNA by Peli1-shRNA lentiviral vector. Expression of Peli1 mRNA in BV2 cells transfected with either Peli1-NC lentiviral vectors or Peli1-KD lentiviral vectors was detected by qRT-PCR. The efficiency of Peli1-KD2 lentiviral vector to knockdown Peli1 mRNA was approximately 79.4% (n=3 replicates, * *P*=0.006, # *P*< 0.001, ns indicating non-significant, vs. Peli1-NC).
