# Supplementary figures and images for: Peli1 Contributions in Microglial Activation, Neuroinflammatory Responses and Neurological Deficits Following Experimental Subarachnoid Hemorrhage
Source: Front Mol Neurosci. 2017 Nov 30;10:398. doi: 10.3389/fnmol.2017.00398 (PMC5714869; doi:10.3389/fnmol.2017.00398)

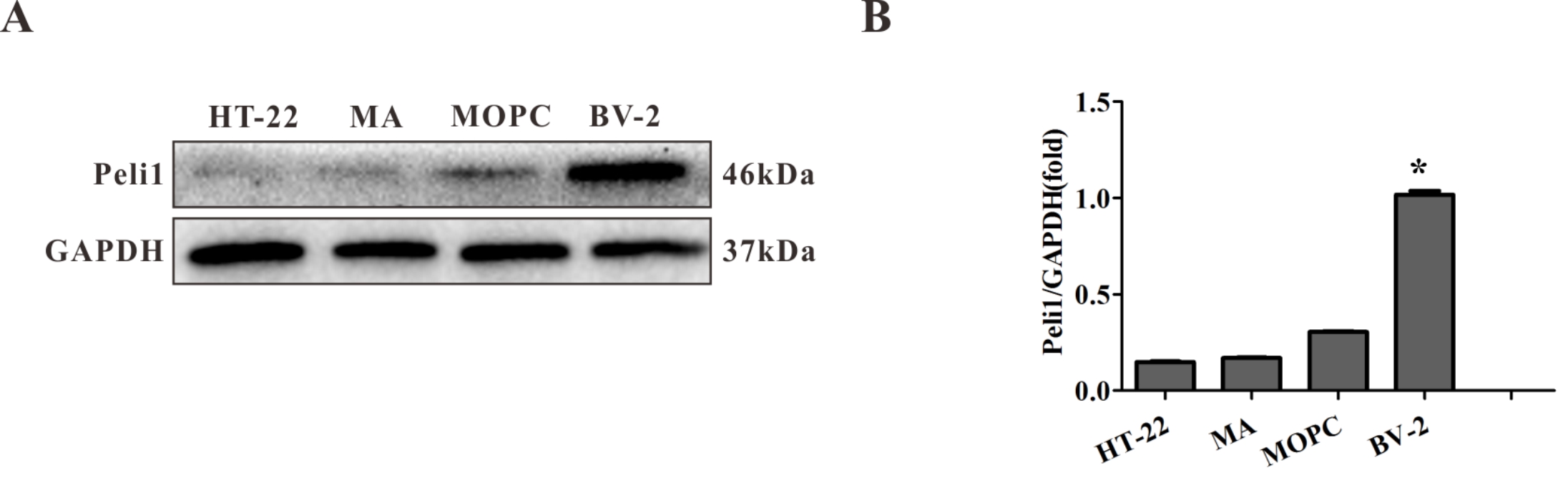

Supplement: Supplementary file 2 [file Image_1.jpeg]

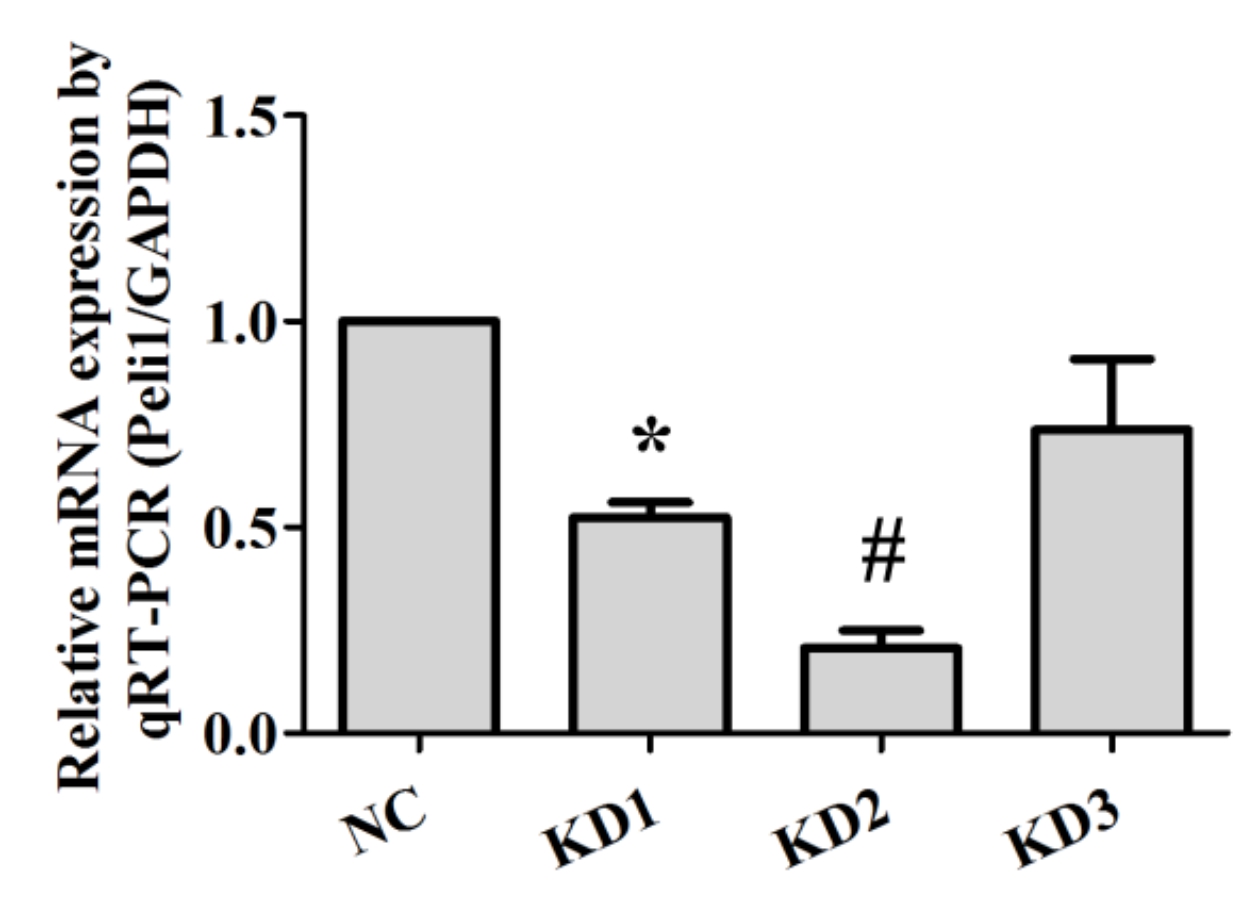

Supplement: Supplementary file 3 [file Image_2.jpeg]
